# Study on Speciation of As, Cr, and Sb in Bottled Flavored Drinking Water Samples Using Advanced Analytical Techniques IEC/SEC-HPLC/ICP-DRC-MS and ESI-MS/MS

**DOI:** 10.3390/molecules24040668

**Published:** 2019-02-14

**Authors:** Wiktor Lorenc, Barbara Markiewicz, Dariusz Kruszka, Piotr Kachlicki, Danuta Barałkiewicz

**Affiliations:** 1 Department of Trace Element Analysis by Spectroscopy Method, Faculty of Chemistry, Adam Mickiewicz University in Poznan, 89b Umultowska Street, 61-614 Poznan, Poland; wlorenc@amu.edu.pl (W.L.); pikbar@interia.pl (B.M.); 2 Institute of Plant Genetics, Polish Academy of Sciences, 34 Strzeszyńska street, 60-479 Poznan, Poland; dkru@igr.poznan.pl (D.K.); pkac@igr.poznan.pl (P.K.)

**Keywords:** high performance liquid chromatography coupled with inductively coupled plasma mass spectrometry, electrospray ionization mass spectrometry, speciation analysis, bottled flavored drinking water samples, method validation

## Abstract

The main aim of the research was to develop a complementary analytical approach consisting of bespoke speciation analysis and non-targeted speciation analysis of As, Sb, and Cr in flavored bottled drinking water samples using HPLC/ICP-DRC-MS and ESI-MS/MS. The scope of two previously developed analytical procedures, (1) multielemental speciation procedure for As^III^, As^V^, Cr^VI^, Sb^III^, and Sb^V^ analysis and (2) arsenic speciation procedure for AsB, As^III^, DMA, MMA, and As^V^ quantification, was extended to the analysis of a new sample type in terms of bespoke speciation analysis. As for the non-targeted speciation, analysis size exclusion chromatography was used with ICP-MS and a complementary technique, ESI-MS/MS, was used for the organic species of As, Sb, and Cr screening. Full validation of procedures 1 and 2 was conducted. Procedure 1 and 2 were characterized with precision values in the range from 2.5% to 5.5% and from 3.6% to 7.2%, respectively. Obtained recoveries ranged from 97% to 106% and from 99% to 106% for procedures 1 and 2, respectively. Expanded uncertainties calculated for procedures 1 and 2 ranged from 6.1% to 9.4% and from 7.4% to 9.9%, respectively. The applicability of the proposed procedures was tested on bottled drinking water samples. Results for the real samples in procedure 1 were in the range from 0.286 ± 0.027 [μg L^−1^] to 0.414 ± 0.039 [μg L^−1^] for As^III^, from 0.900 ± 0.083 [μg L^−1^] to 3.26 ± 0.30 [μg L^−1^] for As^V^, and from 0.201 ± 0.012 [μg L^−1^] to 0.524 ± 0.032 [μg L^−1^] for Sb^V^. Cr^VI^ and Sb^III^ were not detected in any sample. As for procedure 2, results were in the range from 0.0541 ± 0.0053 [μg L^−1^] to 0.554 ± 0.054 [μg L^−1^] for AsB. Results for As^III^ and As^V^ obtained with procedure 2 were in good accordance with results obtained with procedure 1. DMA and MMA were not detected in any sample.

## 1. Introduction

Water is among the most basic elements of the human diet and it is essential for sustaining life and good health. Consumption of bottled drinking water is constantly growing, and recent projections indicate that it is likely to become the most consumed beverage type. Besides bottled drinking water, the consumption of soft drinks, such as flavored and functional bottled drinking waters, has also shown an increasing tendency [1,2,3]. It is well known that polyethylene terephthalate (PET) bottles that are the most commonly used packing material for drinking water can release a notable amount of antimony into drinking water. It was also reported that storage conditions, such as temperature or sunlight exposure and time of storage, can affect the amount of Sb leaching from PET bottles [4,5,6,7,8,9,10,11,12,13,14,15]. Water additives, such as citric acid, or the concentration of total salts in water can affect not only the amount of Sb leaching, but also the chemical forms of Sb present in water, which may form, for example, stable Sb^III^ and Sb^V^ complexes with citric acid [16,17,18,19]. Bottled drinking water stored in PET bottles is also exposed to arsenic contamination [20,21,22,23]. Reimann et al. reported a higher concentration of arsenic in bottled drinking water stored in glass bottles compared with the same water stored in PET bottles [4,6,7]. El-Hadri et al. confirmed the presence of As in soft drinks, such as apple juice or cola [24]. It is not only the material of a bottle that can have an impact on the drinking water quality, but also the additives responsible for its color. Drinking water stored in green bottles may contain higher amounts of chromium in comparison with the same water stored in transparent bottles [4,7]. Chromium has also been found in various soft drinks and juices stored in various containers (PET, glass, steel, or aluminum) [25,26,27]. Not only does the material or color of the bottle determine the leaching of those elements, but also storage conditions, such as the temperature or sunlight exposure and time of storage [28]. The concentration of antimony, arsenic, and chromium in bottled drinking water from the European market ranges from 0.003 [μg L^−1^] to 4.5 [μg L^−1^], from 0.012 [μg L^−1^] to 21.6 [μg L^−1^], and from 0.02 [μg L^−1^] to 28.9 [μg L^−1^] respectively [1,20].

It is well known that the effect of As, Cr, and Sb on human health depends highly on the chemical forms in which these elements occur in drinking water [29,30]. For both arsenic and antimony, their trivalent species are much more toxic than pentavalent. Also, As and Sb as well as Cr^VI^ inorganic compounds were classified as human carcinogens. Contrary to hexavalent chromium, Cr^III^ exhibits no adverse effects on the human body. Complex organic compounds of As and Sb are notably less harmful to humans [11,29,30,31,32]. According to the Drinking Water Directive (Council Directive 98/83/EC of 3 November 1998 on the quality of water intended for human consumption), the maximum acceptable concentration (MAC) for As, Cr, and Sb equals 10 [μg L^−1^], 50 [μg L^−1^], and 5 [μg L^−1^], respectively [33]. Even though the directive applies to a drinking water and flavored waters are formally not included into its scope, those kinds of beverages are often treated as a substitute of mineral water by consumers.

For all the above reasons, the quality control of bottled drinking water is required to determine the concentrations of a wide variety of both inorganic and organic As, Sb, and Cr species. Among different analytical approaches, high performance liquid chromatography coupled with inductively coupled plasma mass spectrometry (HPLC/ICP-MS), equipped with a dynamic reaction cell (DRC), is currently the most frequently applied in the field of speciation analysis. The high diversity of chromatographic separation mechanisms provides various applications of the HPLC technique. In turn, the ICP-MS offers excellent sensitivity and multielemental detection [29]. On the other hand, electrospray ionization mass spectrometry (ESI-MS) seems to be a perfect tool for the determination of the structures of organic compounds potentially present in drinking water samples. In the literature, one can find various applications of ESI-MS in the analysis of organic compounds containing metal atoms, and also arsenic, in food samples [34,35]. New analytical capabilities can be created through the use of both the aforementioned analytical techniques, HPLC/ICP-MS and ESI-MS, in the analysis of a single sample. Such a combination gives more complete information about the sample than the use of each of these techniques independently.

The quality control of the obtained results is always an extremely important issue. This task involves the validation of the analytical procedure, assuring measurement traceability, and estimation of the measurements’ result uncertainty. Validation of analytical procedures concerns the determination of a number of parameters which characterize the procedure; in the speciation analysis procedure, all of the parameters should be determined for each of the analyzed species.

Two analytical methods were previously developed by our team for the speciation analysis of drinking water and used in reals sample analysis: (i) Multielemental speciation procedure for As^III^, As^V^, Cr^VI^, Sb^III^, and Sb^V^ determination [7,18,36,37]; and (ii) arsenic speciation procedure for arsenobetaine (AsB), As^III^, dimethylarsenic acid (DMA, monomethylarsonic acid (MMA), and As^V^ determination [7,18,36,37,38].

The main goal of the present paper was the study on the speciation of As, Cr, and Sb in bottled flavored drinking water samples by two advanced hyphenated techniques of ion exchange chromatography (IEC), size-exclusion chromatography (SEC) HPLC/ICP-DRC-MS, and ESI-MS/MS. The research was carried out in two main areas: (i) Bespoke methods for robust quantification of target elemental analytes, and to conduct the full validation of those methods along with establishing traceability and estimating the uncertainty budget, (ii) screening methods for the identification of different molecular forms of the tested elements in the sample [39]. Bespoke speciation analysis focuses on the quantification of well-known species, which are expected to be found in real samples based on the literature data or previous experiments. On the other hand, non-targeted screening analysis concentrates on searching for new species and their identification.

## 2. Results and Discussion

### 2.1. Validation

**Figure 1:**
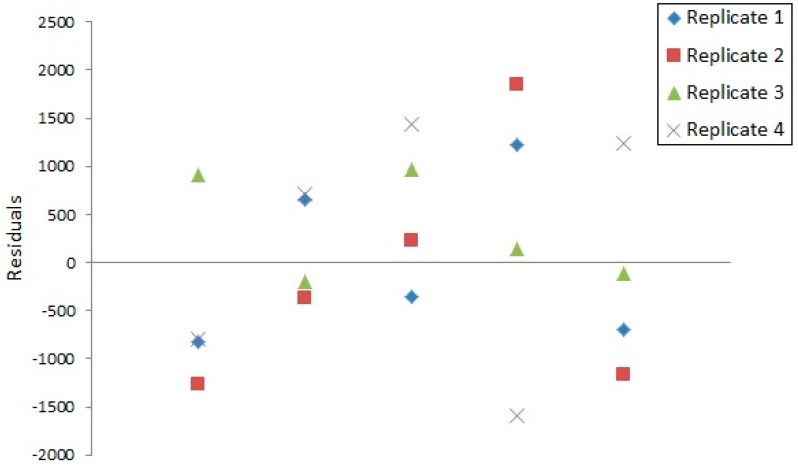
Residuals scatter plot obtained for the DMA calibration plot.

Full validation was conducted for procedure 1 and 2 in which the following parameters were evaluated: Calibration curve linearity, limit of detection (LOD), precision, and trueness. Measurement traceability was provided by the standard addition method to the real samples. Linearity of the calibration curves was confirmed with R^2^ values greater than 0.99 for all analytes. Residual scatter plots obtained for all calibration curves showed a random distribution of residuals around the vertical axis, which is shown on an example residual plot in Figure 1. The F and *p* values calculated based on ANOVA confirmed that a linear relationship exists between variables. LOD values were calculated using the analyte spiked drinking water samples for all analytes in procedure 1 and 2. Found LOD values ranged from 0.046 µg L^−1^ to 0.12 µg L^−1^ for procedure 1 and from 0.053 µg L^−1^ to 0.10 µg L^−1^ for procedure 2. Intermediate precision expressed as CV [%] was estimated using analyte spiked bottled drinking water samples (0.5 µg L^−1^ for procedure 1 and 1 µg L^−1^ for procedure 2) and was found to range from 2.5% to 5.5% for procedure 1 and from 3.6% to 7.2% for procedure 2. Trueness expressed as recovery was estimated using the analyte spiked bottled drinking water samples (0.5 µg L^−1^ for procedure 1 and 1 µg L^−1^ for procedure 2) and ranged from 97% to 109% for procedure 1 and from 99% to 106% for procedure 2. The Student’s *t*-test was performed to verify if the obtained mean recoveries were significantly different from 100% and confirmed that the calculated values were in good agreement with theoretical reference values. Detailed validation results are shown in Table 1.

### 2.2. Uncertainty Budget Estimation

**Figure 2:**
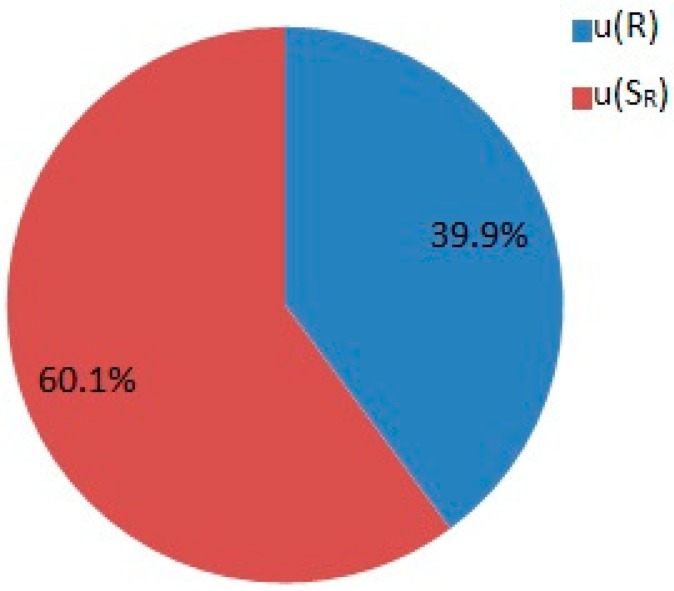
Contribution of the individual components of uncertainty in the overall uncertainty of the AsB concentration.

The measurement uncertainty budget was estimated using the single laboratory validation approach, expanded uncertainty (U) was calculated using k = 2 and expressed as a percentage of the analyte concentration. The calculated expanded uncertainty values for procedure 1 were as follows: 9.4% for As^III^, 9.2% for As^V^, 6.1% for Cr^VI^, 6.6% for Sb^III^, and 6.2% for Sb^V^. For procedure 2, the expanded uncertainty values were: 9.8% for AsB, 9.9% for As^III^, 8.7% for DMA, 9.0% for MMA, and 7.4% for As^V^. The precision component has a greater influence on the combined standard uncertainty than the trueness component for all analytes in procedures 1 and 2. The contributions of the individual components of uncertainty in the overall uncertainty of the AsB concentration are shown in Figure 2. Expanded uncertainty values for all analytes are gathered in Table 1.

### 2.3. Results for Real Samples

All four developed analytical procedures were applied to the analysis of real samples. Five bottled drinking water samples (three brands) were analyzed. Prior to speciation analysis, total concentrations of As, Cr, and Sb were measured using ICP-DRC-MS. Obtained total concentration values were compared with the results of procedure 1 and 2. Based on the differences between the found total As, Cr, and Sb concentrations and the species of those elements determined using procedures 1 and 2, the samples were chosen for analysis using procedures 3 and 4. Two samples (B.1 and C.2) in which the total concentration of As, Cr, and Sb were not in accordance with the concentration of species determined using procedures 1 and 2 were analyzed using procedures 3 and 4. In Procedure 3, the chromatographic peaks for the As, Cr, and Sb compounds eluting from the SEC column were recorded. Procedure 4 was applied to the same samples that were analyzed using procedure 3, which did not provide analytical signals suggesting the presence of organic connections of As, Cr, or Sb. In addition, standard solutions for As (As^V^, MMA, and DMA) were analyzed in order to identify some characteristic fragment ions for potential organic As compounds. Detailed results are described below.

#### 2.3.1. Procedures 1 and 2

Sb^III^, Cr^VI^, DMA, and MMA were not detected in any of the analyzed samples while using procedures 1 and 2. As^III^ was only detected in flavored water samples and As^V^ was detected in two flavored water samples and one unflavored water sample. As^V^ was detected with a notably higher concentration than As^III^ in all those samples. Results obtained with procedure 1 and procedure 2 for the As^III^ and As^V^ concentrations are consistent. AsB was only detected in two flavored water samples. Sb^V^ was detected in one unflavored water sample and one flavored water sample with a pH value considerably closer to 7 than the other two flavored water samples. Additionally, in all three flavored water samples, two additional peaks for chromium were detected, one of which was identified as Cr^III^, although it was not quantified. Detailed results obtained for all samples for the total amounts of As, Cr, and Sb and the results from procedures 1 and 2 are collected in Table 2.

#### 2.3.2. Procedures 3 and 4

Procedures 3 and 4 were applied to two flavored water samples, B.1 and C.2. In the case of procedure 3, in sample B.1, only one chromatographic peak for As was registered, with a retention time of 32 min; no peaks for Cr and Sb were detected. In the sample, C.2, two peaks for As were detected with retention times of 32 and 34 min, three Cr peaks with retention times of 31, 34, and 36 min, and one Sb peak with a retention time of 31 min. In our opinion, the results obtained in procedure 3 may suggest the presence of the organic As, Cr, and Sb compounds; therefore, the samples were subjected to ESI-MS/MS analysis.

Real sample analyses by the application of procedure 4 did not allow us to find any compounds whose fragmentation spectra showed fragments suggesting that these compounds contain arsenic, the reason of which was a low concentration of arsenic and a high amount of sugar in the samples.

During the analysis of As standard solutions (As^III^, As^V^, MMA, and DMA) using procedure 4, the characteristic fragment ions of arsenic compounds were found. During As^III^ standard solution analysis, no molecular ion or any characteristic fragment ions were found. In As^V^ fragmentation spectra, a molecular ion of arsenic acid [H_2_AsO_3_]^−^ was observed with an *m*/*z* value of 140.9163. The most abundant fragment ion found on As^V^ fragmentation spectra with an *m*/*z* value of 122.9048 was assigned to the [AsO_3_]^−^ ion. Another characteristic fragment with an *m*/*z* value of 106.9109 was found and identified as the [AsO_2_]^−^ ion. This fragment ion showed a very low relative abundance although it was characterized by a delta *m*/*z* value equal to −6.054 ppm. In the MMA standard solution fragmentation spectra, a molecular ion with an *m*/*z* value of 138.9366 was found and identified as [CH_3_AsO_3_]^−^. Fragment ions observed on the MMA fragmentation spectra were [H_3_AsO]^−^ with an *m*/*z* value of 123.9125, [CH_2_AsO_2_]^−^ with an *m*/*z* value 120.9258, and [AsO_2_]^−^ with an *m*/*z* value 106.9103. A molecular ion of DMA [(CH_3_)_2_AsO_2_]^−^ with an *m*/*z* value of 136.9574 was found along with characteristic fragments of [CH_3_AsO_2_]^−^ with an *m*/*z* value 121.9344 and [AsO_2_]^−^ with an *m*/*z* value of 106.9109.

In the literature, one can find information about the complexation of antimony by citric acid, also in matrices similar to flavored water samples (juice samples) [16,17,40]. Despite the fact that the tested samples contained citric acid, antimony complexes were not detected. No other antimony or any chromium compounds were found while using procedure 4.

**Figure 3:**
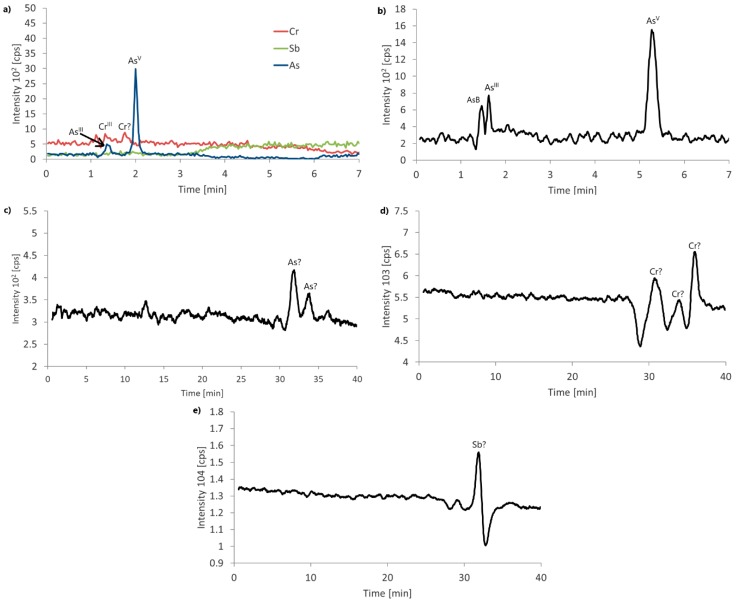
Real sample (C.2) chromatograms for (**a**) procedure 1, (**b**) procedure 2, (**c**) As in procedure 3, (**d**) Cr in procedure 3 and (**e**) Sb in procedure 3.

**Figure 4:**
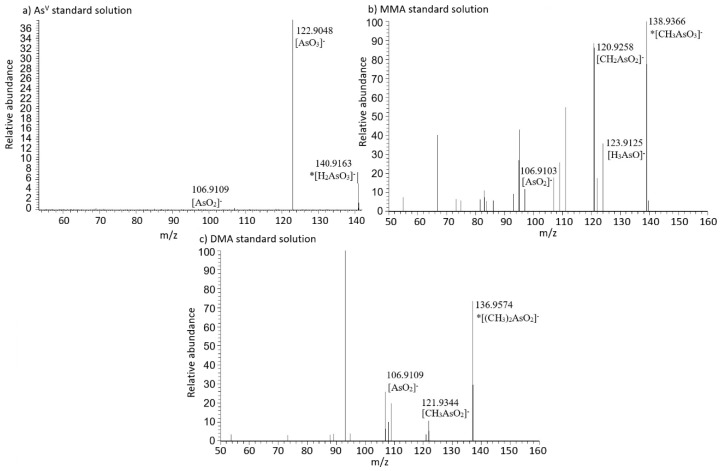
Arsenic species standard solutions fragmentation spectra for (**a**) AsV, (**b**) MMA, (**c**) DMA (* marking denotes a molecular ion).

Detailed results obtained from procedures 3 and 4 are collected in Table 3. Example chromatograms and ESI-MS/MS fragmentation spectra are shown in Figure 3 and Figure 4.

## 3. Materials and Methods

### 3.1. Instrumentation

An Elan DRC II ICP-DRC-MS instrument (PerkinElmer SCIEX, Waltham, MA, United States), was used in the course of the experiments. The DRC with oxygen as a reaction gas was used to remove spectral interferences.

Chromatographic separation was achieved in an HPLC system consisting of a PE Series 200 pump, a column oven, a PE Series 225 auto sampler equipped with a Peltier Cooling Tray, and a PE Series 200 UV/VIS detector (PerkinElmer SCIEX, Ontario, Canada). HPLC and ICP-DRC-MS operating conditions are presented in Table S1 in Supplementary Materials A.

In the course of the ESI-MS/MS experiments, a high resolution mass spectrometer Q-Exactive Orbitrap (Thermo Fisher Scientific, Bremen, Germany) with a heated electrospray source II (HESI-II) was used.

### 3.2. Analytical Procedures

**Figure 5:**
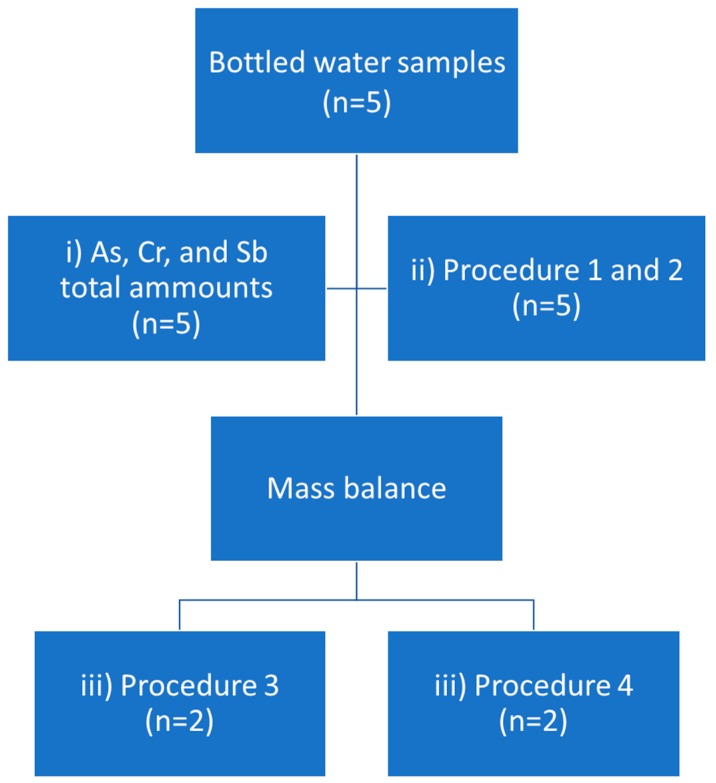
Analytical strategy scheme.

The experiment described in the present paper comprised three steps (i) Total concentration of As, Cr, and Sb quantification, (ii) bespoke speciation analysis for the determination of the target species of As, Cr, and Sb, (iii) non-targeted speciation analysis for the searching of new species of examined elements in the sample and identification of those species. Prior to step (iii), a mass balance, based on the difference in the total concentrations of the target elements and results obtained with procedures 1 and 2, was estimated to decide which of the samples should be analyzed with procedures 3 and 4. Complete analytical strategy was shown as a scheme on Figure 5.

Three analytical procedures for speciation analysis were employed to study the speciation of As, Cr, and Sb in bottled flavored drinking water samples in terms of both inorganic and organic connections of those elements. In addition, an analytical procedure for the identification of organic As, Cr, and Sb species in bottled flavored drinking water samples using the ESI-MS/MS technique was developed. The total amount of As, Cr, and Sb in all samples was analyzed using ICP-DRC-MS. All analytical procedures are described in detail below.

#### 3.2.1. Procedure 1

A previously developed multielemental speciation procedure for As^III^, As^V^, Cr^VI^, Sb^III^, and Sb^V^ analysis using ion-exchange chromatography was used in the analysis of the real samples [7,36]. An anion-exchange HPLC column PRP-X100 (4.6 mm × 150 mm) (Hamilton, Bonaduz, Switzerland) was used to execute the separation of arsenic, chromium, and antimony species. Two eluents were used with an identical composition: 3 mM EDTANa_2_, 36 mM NH_4_NO_3_, and different pH: 4.6 (Eluent A) and 9 (Eluent B). The mobile phase flow was set at 1.2 mL min^−1^, sample injection volume was 100 µL, and column temperature was kept at 25 °C. A gradient elution program was applied: Step 1 (equilibration)—0.5 min of 0% eluent B, step 2—0.1 min of 0% eluent B, step 3—0.1 min of skipping from 0% eluent B to 100% eluent B, step 4—4.3 min of 100% eluent B, step 5—0.1 min of skipping from 100% eluent B to 0% eluent B, step 6—2.9 min of 0% eluent B, step 7 (wash)—6.9 min of 0% eluent B.

#### 3.2.2. Procedure 2

A previously developed arsenic speciation procedure for AsB, As^III^, DMA, MMA, and As^V^ determination was used in the analysis of the real samples [18,37]. A PRP-X100 HPLC anion-exchange column (4.6 mm × 150 mm) (Hamilton, Bonaduz, Switzerland) was used to execute the separation of five arsenic species. A mobile phase composed of 10 mM NH_4_H_2_PO_4_ and 10 mM NH_4_NO_3_ with pH set to 9.2 was used at a 1.2 mL min^−1^ flow rate. The sample injection volume was set at 100 µL and column temperature was kept at 25 °C. An isocratic elution program was employed with a total analysis time of 7 min.

#### 3.2.3. Procedure 3

Multielemental speciation for As, Sb, and Cr analysis using size exclusion chromatography was developed. A Superdex 75 10/300 GL (GE Healthcare, Marlborough, MA, USA) SEC column was used to execute the separation of As, Cr, and Sb species. A mobile phase composed of 50 mM NaH_2_PO_4_ and 30 mM NaCl with pH set to 7.2 was used at a flow rate of 0.55 mL min^−1^. The sample injection volume was set at 100 µL and the column temperature was kept at 25 °C. An isocratic elution program was employed with a total analysis time of 40 min. Due to the high concentration of dissolved salts in the mobile phase (in terms of ICP-MS) and the presence of NaCl, which may lead to nebulizer and cones clogging, the ICP-MS instrument was flushed with 1% HNO_3_ for 5 min before every sample injection [39]. To calibrate the SEC column retention times, a UV/VIS detector (PerkinElmer SCIEX, Ontario, Canada) was used at the same HPLC setting as in the case of ICP-DRC-MS.

**Figure 6:**
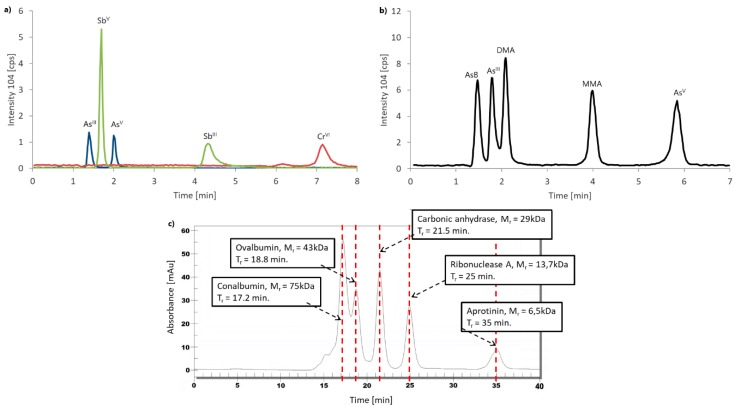
Example chromatograms for: (**a**) Calibration standard for procedure 1, (**b**) calibration standard for procedure 2 and (**c**) molecular weight calibration kit for procedure 3.

Example chromatograms for the calibration standards for procedures 1 and 2 and for the molecular weight calibration kit for procedure 3 are shown in Figure 6.

#### 3.2.4. Procedure 4

The analyses were performed using the high-resolution mass spectrometer, Q-Exactive Orbitrap (Thermo Fisher, Waltham, MA United States), with a heated electrospray source II (HESI-II). Samples were directly injected using a syringe pump at a 5 µL min^−1^ flow rate. The HESI-II source worked in negative ionization mode with an electrospray voltage of −2.5 kV. The experiment was conducted in FullMS-ddMS2 mode for mass range 100—1000 *m*/*z* and resolution was set to 70,000 *m*/*z*. The maximum injection time was 200 ms. The ddMS2 data were recorded at resolution 17,500 and the isolation window was 1 *m*/*z*. The collision energy in the HCD cell was 30 eV. Mass spectra were processed using Xcalibur 2.9 software from ThermoFisher Scientifics.

### 3.3. Sample Collection and Preparation

All developed procedures were applied to real sample analysis. Five non-carbonated bottled drinking water samples (3 brands) were purchased from a local supermarket. In addition to flavored drinking water, an unflavored water sample of the same brand was bought (if available). All the samples were bought in PET bottles. Samples were stored in their original packing, maintaining the storage conditions suggested by the manufacturer and opened directly before the analysis. All samples were bought at the same time and for all the samples, except sample C.1, similar expiry dates were chosen. Samples were analyzed without dilution or any other sample preparation. Detailed characteristics of the samples with their markings are given in Table 4 below.

### 3.4. Chemicals and Reagents

#### 3.4.1. As, Cr, and Sb Working Solutions

All working solutions were prepared from high purity standards. As^III^, As^V^, and Cr^VI^ working solutions were prepared from liquid standards by dilution to the desired concentration with ultrapure water. AsB, DMA, MMA, Sb^III^, and Sb^V^ working solutions were prepared from solid high purity standards by dissolving in ultrapure water. SEC column retention time calibration solutions were prepared from a Low Molecular Weight Gel Filtration Calibration Kit (GE Healthcare, Marlborough, MA, USA) by dissolving in phosphate buffer as suggested by the manufacturer.

#### 3.4.2. Mobile Phases

The mobile phases for all procedures were prepared from high purity reagents using ultrapure water. Mobile phases were always filtered through a membrane filter with a pore size of 0.2 μm immediately after preparation and stored in darkness at 4 °C in plastic bottles. The pH of all mobile phases was set using an electronic pH meter calibrated with three buffer solutions (pH 4.01, 6.87, and 9.18).

#### 3.4.3. Other Reagents

High purity argon (Linde Gas, Kraków, Poland) was used as a nebulizer, auxiliary, and plasma gas for ICP-DRC-MS, also high purity oxygen (Linde Gas, Kraków, Poland) was used as a DRC reaction gas. A Smart Tune Solution—ELAN DRC/PLUS/II (PerkinElmer SCIEX, Ontario, Canada) was used as a daily tuning solution for ICP-DRC-MS. Ultrapure water obtained with a water purification system (TKA Smart2Pure, Niederelbert, Germany) was used in the course of all experiments.

### 3.5. Calibration

The external calibration method was used for procedures 1 and 2. Calibration curves were constructed based on five points over the concentration ranges of: 0.2 µg L^−1^ to 5.0 µg L^−1^ for As^III^ and As^V^, 0.1 µg L^−1^ to 5.0 µg L^−1^ for Sb^V^, 0.5 µg L^−1^ to 5.0 µg L^−1^ for Sb^III^ and Cr^VI^ for procedure 1, and 0.5 µg L^−1^ to 10.0 µg L^−1^ for all species analyzed in procedure 2. The average signal intensity (peak area value) of three replicates for each calibration standard was taken to construct the calibration curves [36,37].

In procedure 3, retention times were calibrated using a Low Molecular Weight Gel Filtration Calibration Kit (GE Healthcare, Marlborough, MA, USA) consisting of five proteins with MW in the range 6500 to 75,000 Da. Retention times were determined using a UV/VIS detector in place of ICP-DRC-MS [35,41].

### 3.6. Analytical Procedure Validation

Full validation was conducted for procedures 1 and 2, while procedures 3 and 4, being only qualitative analysis procedures, were not included in the full validation process. In the validation process, the following parameters were evaluated: Calibration curve linearity, LOD, precision, and trueness. Measurement traceability was provided by applying the standard addition method to real samples [42,43].

#### 3.6.1. Linearity

To evaluate the quality of the obtained calibration curves, coefficient of determination (R^2^) values were calculated as well as residual analysis and a significance F test (based on the analysis of variance, ANOVA) were performed. Residual analysis is based on a visual analysis of a residual scatter plot in which residuals are shown on the vertical axis and the independent variable is shown on the horizontal axis. Residuals shown on a graph should be randomly distributed around the horizontal axis and should not exhibit any trends. The F value of the significance test, calculated using the regression mean square value and the residual mean square value, is then compared with the critical F value and, along with the p-value associated with the calculated F-statistic, can confirm that a linear relationship between variables exists [44,45].

#### 3.6.2. Limits of Detection

To determine LOD values, for each procedure, a series of 9 analyte spiked drinking water samples were prepared (diluted 4 times to obtain a drinking water matrix, without analytes). A quantifiable amount of analytes, close to the expected LOD value (0.2 µg L^−1^), were added and all samples were analyzed using the presented analytical procedures. LOD values were calculated as three times the standard deviation of the measurement results [46,47].

#### 3.6.3. Precision

Precision was evaluated under intermediate precision conditions—Measurements were conducted over the course of several days. An evaluation of precision was determined according to the recommendation of the International Conference on Harmonization. To estimate the precision for each procedure, a series of 9 independent bottled drinking water samples with analyte addition (0.5 µg L^−1^ for procedure 1 and 1 µg L^−1^ for procedure 2) were analyzed using the presented analytical procedures (with three replicates for each sample). Calculations were based on the measured signal area. Intermediate precision was expressed as a coefficient of variation (CV) [%] [48].

#### 3.6.4. Trueness

The standard addition method was employed to assess the trueness of both analytical procedures. Similarly to the precision evaluation, a series of 9 independent bottled drinking water samples with analyte addition for each procedure were employed. Each of the samples was spiked with authentic standards (0.5 µg L^−1^ for procedure 1 and 1 µg L^−1^ for procedure 2) and analyzed using the presented analytical procedures (with three replicates for each sample). The recovery was calculated according to guidelines recommended by IUPAC based on the measured signal area for each of the analytes [49]. The Student’s t test was applied to verify if the obtained recovery values were significantly different from 100% [49].

### 3.7. Measurement Uncertainty

In the presented paper, the uncertainty budget estimation was conducted using the single laboratory validation approach. The single laboratory validation method for uncertainty estimation is based on the data obtained during the validation process. To estimate the uncertainty budget, parameters influencing the measurement uncertainty of the analytical result were grouped into precision and trueness components. The overall analytical method precision was estimated using intermediate precision values. The use of intermediate precision conditions allows all sources of uncertainty related to volumetric measuring equipment, influences of environmental conditions, repeatability, and the drift of an analytical instrument to be considered. The standard uncertainty of the precision of the method was calculated using relative standard deviation values. The trueness of the method was estimated based on the spiked samples of drinking water as mentioned before. The Student’s t-test, performed to verify if the obtained mean recoveries were significantly different from 100%, confirmed that the calculated recoveries were in good agreement with the theoretical reference. The combined standard uncertainty of an analyte concentration was calculated according to the Equation:

$$u_{c}\left(C_{a}\right)=C_{a}\sqrt{u^{2}\left(s_{R}\right)+u^{2}(\bar{R`)}}$$

where u (S_R_) denotes the standard uncertainty of precision and u $(\bar{R`)}$ denotes the standard uncertainty of recovery [50,51].

## 4. Conclusions

In the present work, we developed a complementary analytical approach consisting of bespoke speciation analysis and non-targeted speciation analysis of As, Sb, and Cr in bottled drinking water samples using HPLC/ICP-DRC-MS and ESI-MS/MS.

In terms of bespoke speciation analysis, two analytical procedures for the determination of various inorganic (As^III^, As^V^, Sb^III^, Sb^V^, Cr^III^, and Cr^VI^) as well as organic (AsB, MMA, DMA) species in bottled drinking water samples were employed. Arsenic was detected in tested samples with the highest concentration. Also, arsenic species were the most prevalent in the analyzed samples. As^V^ was detected in higher concentrations than As^III^ in all samples. AsB was only detected in flavored water samples. Sb^III^ was not detected in any of the samples and Sb^V^ was only detected in unflavored water samples and one flavored water sample. Cr^VI^ was not detected in any of the samples; however, Cr^III^ and an unidentified Cr species were detected in flavored water samples. Results obtained for real samples analyzed during this experiment do not exceed permissible concentrations in accordance to the Drinking Water Directive (Council Directive 98/83/EC of 3 November 1998 on the quality of water intended for human consumption).

The reliability of the developed analytical procedures was assessed through traceability assurance and validation of the analytical procedures. The uncertainty budget was estimated using the single laboratory validation approach. Obtained validation parameters confirmed the applicability of the presented procedures for their intended purpose.

In terms of the non-targeted speciation analysis, analytical procedures meant for the detection of complex organic compounds and confirming that they contain arsenic were developed. No complex organic compounds of arsenic were detected in real samples. Characteristic fragment ions containing arsenic were found in the fragmentation spectra of As^V^, MMA, and DMA standard solutions. Identified fragment ions may be used in the future for organic compounds of arsenic identification in real samples.

## Figures and Tables

**Table 1:** Analytical procedures validation results.

Analytical Procedure Parameter	Analyte				
Procedure 1	As^III^	As^V^	Cr^VI^	Sb^III^	Sb^V^
Retention time [min]	1.4	2.0	7.2	4.4	1.7
Linear range [µg L^−1^]	0.2–5.0	0.2–5.0	0.5–5.0	0.5–5.0	0.1–5.0
Determination coefficient range	0.997–0.9999	0.996–0.9999	0.998–1.0000	0.999–0.9999	0.997–1.0000
LOD [µg L^−1^]	0.058	0.051	0.12	0.090	0.046
Recovery at 0.5 µg L^−1^ [%]	101	99	109	97	103
Intermediate precision [%]	4.3	5.5	2.5	3.1	5.5
Expanded uncertainty (k = 2) [% of analyte concentration]	9.4	9.2	6.1	6.6	6.2
Procedure 2	AsB	As^III^	DMA	MMA	As^V^
Retention time [min]	1.5	1.8	2.1	4.0	5.8
Linear range [µg L^−1^]	0.5–10.0	0.5–10.0	0.5–10.0	0.5–10.0	0.5–10.0
Determination coefficient range	0.995–0.999	0.995–0.9999	0.996–0.9999	0.997–0.9999	0.996–0.9999
LOD [µg L^−1^]	0.054	0.081	0.053	0.10	0.080
Recovery at 1 µg L^−1^ [%]	106	99	102	104	100
Intermediate precision [%]	3.6	4.8	3.9	7.2	4.4
Expanded uncertainty (k = 2) [% of analyte concentration]	9.8	9.9	8.7	9.0	7.4
Procedure 3	Conalbumin 75 kDa	Ovalbumin 43 kDa	Carbonic anhydrase 29 kDa	Ribonuclease A 13.7 kDa	Aprotinin 6.5 kDa
Retention Time [min]	17.2	18.8	21.5	25.0	35.0

* LOD—Limit of Detection.

**Table 2:** Real samples’ measurement results for the total amounts of As, Cr, and Sb and measurement results of procedures 1 and 2.

Sample	Total Amounts (Concentration of Analytes ± U [μg L^−1^])				
	As	Cr	Sb		
A.1	0.0078 ± 0.0011	0.0115 ± 0.0014	0.1857 ± 0.0059		
A.2	0.318 ± 0.020	0.0740 ± 0.0066	0.503 ± 0.015		
B.1	8.37 ± 0.52	0.525 ± 0.027	0.643 ± 0.037		
C.1	0.933 ± 0.014	0.3225 ± 0.0012	0.2541 ± 0.0019		
C.2	2.446 ± 0.096	0.1785 ± 0.0015	0.2287 ± 0.0012		
	**Procedure 1 (Concentration of Analytes ± U [μg L^−1^])**				
	**As^III^**	**As^V^**	**Sb^III^**	**Sb^V^**	**Cr^VI^**
A.1	<LOD	<LOD	<LOD	0.201 ± 0.012	<LOD
A.2	0.327 ± 0.031	<LOD	<LOD	0.524 ± 0.032	<LOD
B.1	0.414 ± 0.039	3.26 ± 0.300	<LOD	<LOD	<LOD
C.1	<LOD	0.900 ± 0.083	<LOD	<LOD	<LOD
C.2	0.286 ± 0.027	1.68 ± 0.15	<LOD	<LOD	<LOD
	**Procedure 2 (Concentration of Analytes ± U [μg L^−1^])**				
	**AsB**	**As^III^**	**DMA**	**MMA**	**As^V^**
A.1	<LOD	<LOD	<LOD	<LOD	<LOD
A.2	<LOD	0.317 ± 0.031	<LOD	<LOD	<LOD
B.1	0.554 ± 0.054	0.432 ± 0.043	<LOD	<LOD	3.16 ± 0.234
C.1	<LOD	<LOD	<LOD	<LOD	0.957 ± 0.071
C.2	0.0541 ± 0.0053	0.302 ± 0.030	<LOD	<LOD	1.72 ± 0.13

* LOD—Limit of Detection.

**Table 3:** Measurement results for procedures 3 and 4.

Sample	Procedure 3		
	As	Cr	Sb
B.1	One peak: ** t_R1_ = 32 min	-	-
C.2	Two peaks: t_R1_ = 32 min t_R2_ = 34 min	Three peaks: t_R1_ = 31 min t_R2_ = 34 min t_R3_ = 36 min	One peak: t_R1_ = 31 min
	**Procedure 4**		
	**Ion Formula**	***m*/*z*_exp_ (*m*/*z*_theo_)**	
As^V^ standard solution	* [H_2_AsO_3_]^−^	140.9163 (140.9161)	
	[AsO_3_]^−^	122.9048 (122.9054)	
	[AsO_2_]^−^	106.9109 (106.9104)	
MMA standard solution	* [CH_3_AsO_3_]^−^	138.9366 (138.9368)	
	[H_3_AsO]^−^	123.9125 (123.9129)	
	[CH_2_AsO_2_]^−^	120.9258 (120.9260)	
	[AsO_2_]^−^	106.9103 (106.9101)	
DMA standard solution	* [(CH_3_)_2_AsO_2_]^−^	136.9574 (138.9574)	
	[CH_3_AsO_2_]^−^	121.9344 (121.9338)	
	[AsO_2_]^−^	106.9109 (106.9104)	

* Molecular Ion. ** t_R_—Retention time.

**Table 4:** Sample characteristics.

Sample	Flavor	pH	Mineralization [mg L^−1^]	Bottle Color	Composition Stated by Manufacturer
A.1	Unflavored	6.52	1670.9	Colorless	-
A.2	Lemon	5.91	1670.9	Light Blue	Mineral water, natural lemon flavor with other natural flavors
B.1	Apple	3.42	775.2	Green	Mineral water, sugar, apple juice from concentrate, grape juice from concentrate, flavor, citric acid, ascorbic acid
C.1	Unflavored	7.05	-	Light Blue	-
C.2	Strawberry	3.47	-	Light Blue	Mineral water, glucose-fructose syrup, sugar, citric acid, natural strawberry flavor with other natural flavors, sweeteners (acesulfame K, sucralose)

## Supplementary Materials

The supplementary materials are available online.

## List of Abbreviations

PET—Polyethylene terephthalate; MAC—Maximum acceptable concentration; HPLC—High performance liquid chromatography; ICP-MS—Inductively coupled plasma mass spectrometry; DRC—Dynamic reaction cell; ESI-MS—Electrospray ionization mass spectrometry; AsB—Arsenobetaine; DMA—Dimethylarsenic acid; MMA—Monomethylarsonic acid; IEC—Ion exchange chromatography; SEC—Size-exclusion chromatography; LOD—Limit of detection; CV—Coefficient of variation.
