# Supplementary material for: Study on Speciation of As, Cr, and Sb in Bottled Flavored Drinking Water Samples Using Advanced Analytical Techniques IEC/SEC-HPLC/ICP-DRC-MS and ESI-MS/MS
Source: Molecules. 2019 Feb 14;24(4):668. doi: 10.3390/molecules24040668 (PMC6412991; doi:10.3390/molecules24040668)
Supplement: Supplementary file 1 [file molecules-24-00668-s001.pdf]

Table S1. Operating parameters for HPLC/ICP-DRC-MS and ESI-MS/MS

		Parameter	Setting
ICP-MS		Instrument	PE Sciex ELAN 6100 DRC II
		RF Power	1100-1200 W
		Nebulizer gas (Ar) flow rate	0.87-0.93 L min^{-1}
		Auxilary gas (Ar) flow rate	1.20 L min^{-1}
		Plasma gas (Ar) flow rate	16 L min^{-1}
		Sampler and skimmer cones	Pt
		Lens voltage	7.0 - 9.5 V
		Detector mode	Dual
		Data collection mode	^{91}AsO, ^{52}Cr, ^{121}Sb
		Scan mode	Peak hopping
		DRC gas (O_2) flow rate	0.55 mL min^{-1}
		Rpq	0.55
		Rpa	0
HPLC		Instrument	PE series 200 HPLC pump<br>PE series 225 HPLC autosampler<br>PE series 200 column oven<br>PE series 200 UV detector
	Procedure 1	Column	Hamilton PRP-X100
		Elution	Gradient
		Mobile phase content	EDTA disodium salt, ammonium nitrate
		Concentration of mobile phase	0.003 mol L^{-1} $EDTANa_2$,<br>0.036 mol L^{-1} NH_4NO_3
		pH of mobile phase	Eluent A: 4.6; Eluent B: 9.0
		Mobile phase flow rate	1.2 mL min^{-1}
		Injection volume	100 µL
		Column temperature	25°C
	Procedure 2	Column	Hamilton PRP-X100
		Elution	Isocratic
		Mobile phase content	ammonium dihydrogen phosphate, ammonium nitrate
		Concentration of mobile phase	0.01 mol L^{-1} $NH_4H_2PO_4$,<br>0.01 mol L^{-1} NH_4NO_3
		pH of mobile phase	9.2
		Mobile phase flow rate	1.2 mL min^{-1}
		Injection volume	100 µL
		Column temperature	25°C
	Procedure 3	Column	Superdex 75 10/300 GL SEC
		Elution	Isocratic
		Mobile phase content	sodium dihydrogen phosphate, sodium chloride
		Concentration of mobile phase	0.05 mol L^{-1} $NH_4H_2PO_4$,<br>0.03 mol L^{-1} NaCl
		pH of mobile phase	5.2
		Mobile phase flow rate	0.55 mL min^{-1}
		Injection volume	100 µL
		Column temperature	25°C

ESI-MS/MS	Procedure 4	Instrument	Q-Exactive Orbitrap MS Thermo Fisher
		Syringe pump flow rate	5 μl min^{-1}
		Ion source	HESI -II
		Sheath gas (N_2) flow rate	15 units
		Auxilary gas (N_2) flow rate	5 units
		Auxilary gas temperature	120 °C
		Electrospray Voltage	-2.5 kV
		Ion transfer tube temperature	250 °C
		S-lens RF	50
		Collision energy	30 eV

*ICP-MS – inductively coupled plasma spectrometry; HPLC - high performance liquid chromatography; ESI-MS/MS - electrospray ionization tandem mass spectrometry; DRC – dynamic reaction cell; Rpa - rejection parameter a; Rpq - rejection parameter q.
